# Clinical potential of fluid-based biomarkers for recurrent implantation failure: a systematic review

**DOI:** 10.3389/fendo.2026.1854111

**Published:** 2026-06-12

**Authors:** Mojtaba Moradi, Leilani L. Santos, Wei Zhou, Ellen Menkhorst, Evdokia Dimitriadis

**Affiliations:** 1 Department of Obstetrics, Gynecology and Newborn Health, University of Melbourne, Parkville, VIC, Australia; 2 Gynaecology Research Centre, Royal Women’s Hospital, Parkville, VIC, Australia

**Keywords:** biomarker, blood, endometrial receptivity, menstrual fluid, repeated implantation failure, uterine fluid

## Abstract

**Background:**

Recurrent implantation failure (RIF) remains a significant challenge in reproductive medicine, with diagnostic approaches that are limited and widely debated. This systematic review evaluates whether fluid-based biomarkers from blood, uterine fluid, and menstrual fluid can provide more accessible and clinically informative alternatives.

**Methods:**

A systematic search of MEDLINE, Embase, Scopus, and Web of Science was conducted (2015–2025). Study quality was assessed using QUADAS-2 for diagnostic studies and PROBAST for prognostic studies. Due to substantial methodological heterogeneity, meta-analysis was not performed. Findings were synthesized narratively, and diagnostic performance was summarized using forest plots of reported AUC values without statistical pooling.

**Results:**

From 3,105 records, 18 studies met the inclusion criteria. The evidence base was dominated by blood-based biomarkers (n = 17). Multi-marker models consistently demonstrated higher diagnostic performance than single analytes, with immune-cell profiling panels and cytokine–clinical parameter models achieving AUC values up to 0.94. In contrast, individual biomarkers generally showed lower performance (e.g., lymphocyte count, AUC = 0.577). Prognostic evidence for blood biomarkers was limited, although plasma D-dimer (AUC ≈ 0.81) and immune-cell profiling models (AUC ≈ 0.72) showed potential for predicting pregnancy outcomes. Only one study evaluated uterine fluid biomarkers, identifying a four-protein panel with promising diagnostic performance, while no eligible studies assessed menstrual fluid.

**Conclusion:**

Fluid-based biomarkers, particularly multi-marker models, may have diagnostic potential in RIF; however, current evidence remains insufficient to support routine clinical implementation. Future research should prioritize prospective validation of blood-based multi-marker models in standardized, euploidy-controlled cohorts, alongside greater investigation of biologically proximal uterine and menstrual biofluids.

**Systematic review registration:**

https://www.crd.york.ac.uk/PROSPERO/, identifier CRD420251068643.

## 1 Introduction

Recurrent implantation failure (RIF) is a poorly defined clinical condition generally characterized by the absence of successful embryo implantation after multiple embryo transfers in ART cycles (1–3). Despite advancements in ART, RIF remains a critical barrier in reproductive medicine as a key cause of recurrent ART treatment failure (1, 2, 4, 5). It is estimated that RIF may affect up to 15% of women undergoing IVF procedures (6, 7).

Embryo implantation is a multifaceted process demanding synchronization between a viable blastocyst and a receptive endometrium through the effective interaction of embryonic and maternal components (2, 5). Although advances in preimplantation genetic testing have improved the selection of euploid (chromosomally normal) embryos, implantation failure still occurs, indicating that embryo quality alone does not determine outcomes (8, 9). The endometrium is now recognized as a dynamic and selective biosensor that integrates hormonal, immune, and molecular signals to regulate embryo recognition, implantation, and early placentation (10). Endometrial dysfunction, including altered receptivity, displaced window of implantation (the limited period of uterine receptivity, typically days 20–24 of a natural cycle, during which the endometrium permits embryo attachment and implantation), and immune dysregulation, have increasingly been implicated as a potential contributor to RIF (2). However, existing diagnostic approaches, including histological dating, transvaginal ultrasound, hormone assays, and endometrial transcriptomic tests, have demonstrated variable clinical utility, may require invasive biopsy procedures, and have shown uncertain benefit in improving live birth rates (11–14).

Liquid biopsy approaches using blood, uterine fluid, and menstrual fluid have emerged as promising strategies for the non-invasive assessment of biomarkers reflecting systemic and local endometrial environments (15, 16). Blood biomarkers capture systemic hormonal and immune influences, while uterine fluid contains embryo–maternal signaling molecules that more directly reflect the implantation niche (17–19). Menstrual fluid is a unique biological sample that encompasses shed tissue, extracellular vesicles, and diverse cell populations, including stromal, epithelial, progenitor, and regulatory immune cells involved in implantation (20). Despite growing research, the diagnostic and prognostic performance of these fluid-based biomarkers in RIF remains unclear.

To our knowledge, no systematic review has comparatively evaluated biomarkers across multiple biofluids, blood, uterine fluid, and menstrual fluid, in the context of RIF. The present systematic review therefore aims to critically assess the available evidence on fluid-based biomarkers for diagnosing RIF and predicting treatment outcomes. By synthesizing current diagnostic and prognostic data, this review evaluates whether these minimally invasive biomarkers may provide more accessible and clinically informative tools for patient stratification. In addition, the review identifies key gaps in the existing literature to inform future research and support the development of more evidence-based diagnostic approaches.

## 2 Methods

### 2.1 Registration

This systematic review was conducted according to the Preferred Reporting Items for Systematic Reviews and Meta-analysis (PRISMA) guidelines. The protocol was registered in PROSPERO (registration number: CRD420251068643).

### 2.2 Search strategy

A systematic literature search of MEDLINE, Embase, Scopus, and Web of Science was conducted for studies published from January 1, 2015, to February 11, 2025. The search strategy was structured around three core concepts, (i) condition (recurrent implantation failure and endometrial receptivity), (ii) biological fluid type (blood, uterine fluid, and menstrual fluid), and (iii) biomarkers, combining controlled vocabulary (e.g., MeSH and Emtree terms) with free-text keywords and their synonyms using Boolean operators (AND/OR). The full search strategy is provided in **Supplementary 1**. No language restrictions were applied in the initial search. Duplicate records were removed using Covidence software. The database search was supplemented by manually screening the reference lists of included articles and relevant reviews for additional records.

### 2.3 Eligibility criteria

Eligible studies included peer-reviewed articles published in English that evaluated fluid-based biomarkers (from blood, uterine fluid, or menstrual fluid) in women with RIF. The search was intentionally focused on these three biofluids due to their direct biological relevance to the implantation process. RIF was defined in this review as a history of at least two failed embryo transfers, a broad and inclusive definition chosen to maximize inclusivity and reflect real-world clinical heterogeneity. Given the profound heterogeneity in historical RIF definitions (5), using a stricter criterion would have excluded a large portion of the available evidence and limited the comprehensiveness of this review. We included all study designs that assessed either diagnostic accuracy (distinguishing women with RIF from controls) or prognostic accuracy (predicting clinical pregnancy or live birth within the RIF cohort) and reported performance metrics such as area under the receiver operating characteristic curve (AUC; a measure of diagnostic accuracy ranging from 0.5, indicating no discriminatory ability, to 1.0, indicating perfect discrimination), sensitivity, or specificity.

The selection of studies was performed independently by two reviewers (M.M., L.S.) using Covidence. The first round of selection was based on screening titles and abstracts. A full-text review was then performed independently by two reviewers (M.M., W.Z.). Any disagreements between reviewers were resolved through discussion to reach a consensus. If a consensus could not be reached, a third author (E.D.) made the final decision. Reasons for excluding studies at the full-text review stage were recorded and are presented in the PRISMA flow diagram.

To ensure the integrity of the review, we performed a rigorous trustworthiness screening of all potentially eligible studies using the TRACT checklist (21) and criteria proposed by the Fertility and Sterility editorial board (22). We assessed studies for fatal flaws, including implausible recruitment numbers (e.g., perfect group balancing in observational settings), mathematical impossibilities in baseline characteristics, and lack of verifiable ethical governance. Studies failing this screening were excluded to minimize the inclusion of potentially unreliable data.

### 2.4 Data extraction

Two reviewers (M.M., E.M.) independently extracted data using a standardized form, capturing study characteristics, participant demographics, RIF definitions, biomarker details, performance metrics and were cross-verified.

### 2.5 Quality assessment

The risk of bias for diagnostic accuracy studies was independently assessed by two reviewers (M.M., L.S.) using the Quality Assessment of Diagnostic Accuracy Studies 2 (QUADAS-2) tool. For studies evaluating prognostic accuracy, the Prediction model Risk of Bias Assessment Tool (PROBAST) was used. Disagreements during data extraction or bias assessment were resolved by consensus.

### 2.6 Data synthesis

**Figure 1:**
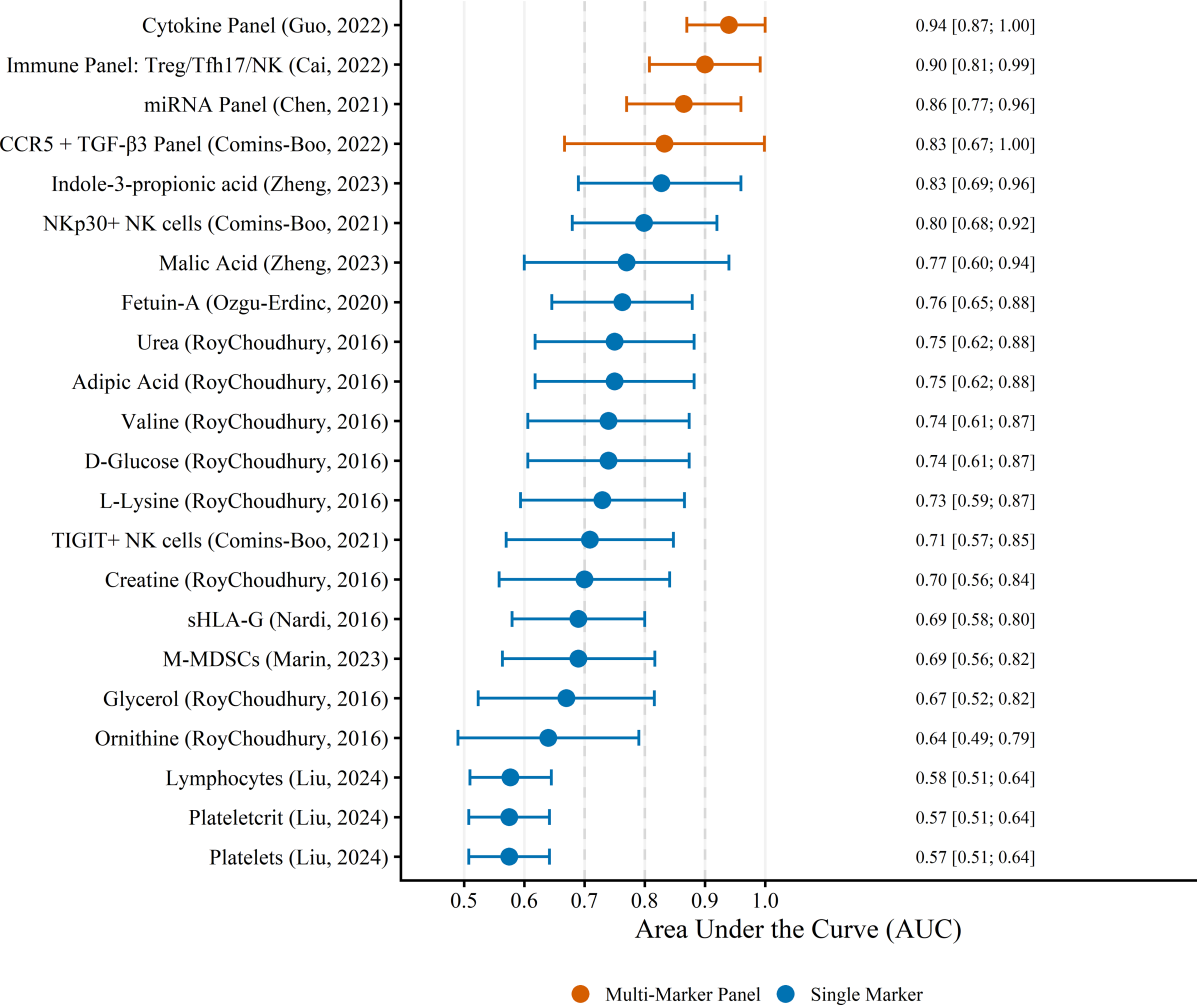
Forest plot of diagnostic accuracy (AUC) for blood-based biomarkers of recurrent implantation failure (RIF). Studies are ranked by AUC to illustrate the comparative performance of different biomarker strategies. Orange circles represent multi-marker panels; Blue circles represent single biomarkers. Error bars indicate 95% Confidence Intervals (CIs).

Given the significant clinical and methodological heterogeneity across the included studies, particularly regarding RIF definitions and control group selection, a quantitative meta-analysis was precluded. Instead, a narrative synthesis of the evidence was performed, structured by biofluid source and clinical utility (diagnostic vs. prognostic). To visualize diagnostic performance, structured forest plots were generated using R statistical software (R Foundation for Statistical Computing, Vienna, Austria). Studies were stratified by biomarker type (single analyte vs. multi-marker panel) and ranked by Area Under the Curve (AUC). For studies where the AUC was reported without 95% Confidence Intervals (CIs), variance estimates were derived using the non-parametric method of Hanley and McNeil (1982) based on the reported AUC and sample sizes (23). These derived intervals were utilized solely to visualize precision in the forest plots (**Figure 1**) and are distinguished from author-reported data in the summary tables.

## 3 Results

### 3.1 Study selection

**Figure 2:**
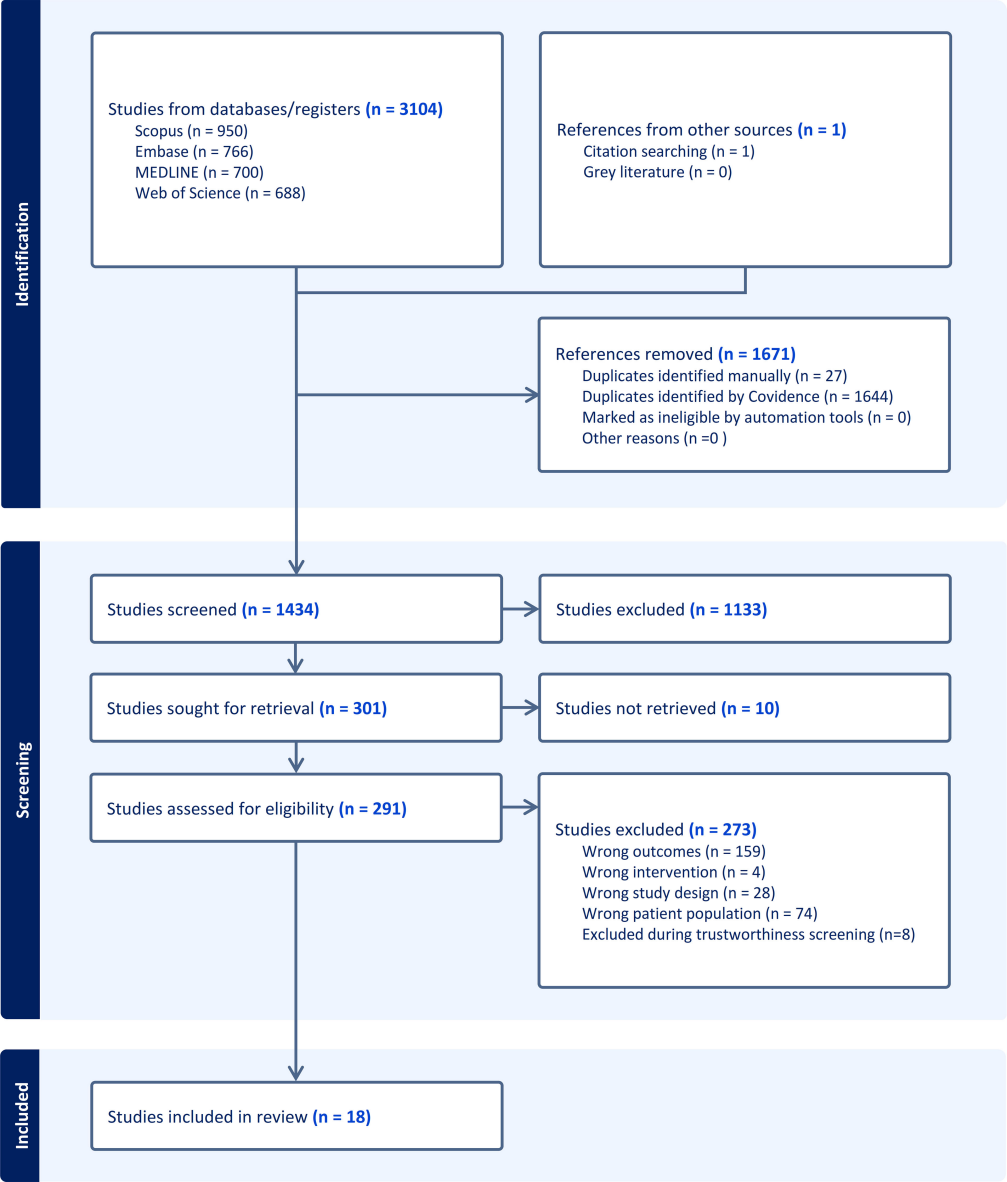
PRISMA flowchart of the study selection process.

Literature searching retrieved 3,105 records. After the removal of duplicates, 1,434 publications were screened on their titles and abstracts, resulting in the selection of 291 articles for full-text screening. We excluded 273 citations during full-text review, resulting in a total of 18 studies included in the review (**Figure 2**).

### 3.2 Characteristics of the included studies

This review evaluated 18 studies involving 13 case-control and 5 cohort studies conducted across 8 countries: Brazil, China, Estonia, India, Poland, Spain, Sweden, and Turkey. The study populations consisted of participants diagnosed with RIF compared against control groups, though the definition of controls varied from fertile women with natural conceptions to infertile patients who achieved live birth after ART treatment. 17 studies evaluated biomarkers derived from blood (serum or plasma), while one study utilized uterine fluid as the biological source for biomarker analysis. Key study characteristics are summarized in **Tables 1**–**3**.

**Table 1:** Summary of studies evaluating immunological biomarkers for the diagnosis of RIF.

Study	Study design	Population	RIF definition & control group	Embryo ploidy status & selection	Fluid & sample timing	Biomarker(s) & assay method	Key findings & clinical utility statistics
Liu et al., 2024; China (24)	Retrospective Case-control study	N=313 (150 RIF/163 Control)	RIF: ≥3 failed ETs with high-quality embryos, or ≥10 embryos transferred total. Control: Age-matched; male-factor ICSI; live birth after 1st ET.	Not assessed; morphology only.	Blood; any day of menstrual cycle.	Platelets (PLT), Plateletcrit (PCT), Lymphocytes; Cytoanalysis Assay: Complete Blood Count (CBC)	Finding: Platelet (PLT), plateletcrit (PCT), and lymphocyte counts were significantly lower in the RIF group compared to the control group. Diagnostic Utility: PLT: AUC: 0.575 (95% CI: 0.508-0.642); Sens: 48.6%; Spec: 66.4%. PCT: AUC: 0.575 (95% CI: 0.508-0.642); Sens: 77%; Spec: 38.9%. Lymphocyte: AUC: 0.577 (95% CI: 0.510-0.645); Sens: 49.3%; Spec: 71%.
Marin et al., 2023; Spain (25)	Retrospective Case-control study	N=108 (41 RIF/27 Healthy Controls) Note: The study also included a cohort of 40 women with Recurrent Pregnancy Loss (RPL).	RIF: ≥4 good-quality embryos in ≥3 cycles; age <40. Control: Proven fertility (≥2 children); no miscarriage history.	Not assessed; morphology only.	Blood; any day of menstrual cycle.	Monocytic-Myeloid Derived Suppressor Cells (M-MDSCs); Assay: Multiparametric flow cytometry.	Finding: Circulating M-MDSC levels were significantly higher in the RIF group compared to healthy controls. Diagnostic Utility: AUC: 0.690 (95% CI: 0.564-0.817); Sens: 75.6%; Spec: 57.7% (at a cutoff of >6.1%).
Comins-Boo et al., 2021; Spain (29)	Retrospective Case-control study	N=114 (25 RIF/31 Control) Note: The study also included a cohort of 58 RPL patients.	RIF: ≥4 good-quality embryos in ≥3 cycles; age <40. Control: Proven fertility (≥2 children); no miscarriage history.	Not assessed; morphology only.	Blood; any day of menstrual cycle.	NK cell surface markers (NKp30, TIGIT) on cytotoxic NK cells (CD56 dim CD16 pos/neg); Assay: Multiparametric flow cytometry.	Finding: The expression of NKp30 and TIGIT on cytotoxic NK (cytNK) cells was significantly higher in the RIF group compared to healthy controls. Diagnostic Utility: NKp30+ cytNK: AUC: 0.80 [0.68–0.92]* Sens: 87%; Spec: 55%. TIGIT+ cytNK: AUC: 0.71 [0.57–0.85]* Sens: 46%; Spec: 77%.
Cai et al., 2022; China (28)	Retrospective Case-control study	N=41 (21 RIF/20 Control)	RIF: ≥3 failed IVF cycles; 1–2 high-grade embryos/cycle. Control: Tubal-factor; successful IVF pregnancy; child >1 year.	Not assessed; morphology only (≥3BB).	Blood; mid-luteal phase.	Lymphocyte subsets including T follicular helper cells (Tfh1, Tfh2, Tfh17), regulatory T cells (Tregs), and early inhibitory Natural Killer (NK) cells; Assay: Flow cytometry	Finding: The RIF group had significantly lower percentages of regulatory T cells (Tregs) and early inhibitory NK cells, and a significantly higher percentage of T follicular helper 17 (Tfh17) cells compared to controls. Diagnostic Utility: For distinguishing RIF from fertile controls using a combined panel of 3 biomarkers (Treg, Tfh17, and early inhibitory NK cells): AUC: 0.900 (95% CI: 0.808-0.992); Sens: Not Reported; Spec: Not Reported.
Comins-Boo et al., 2022; Spain	Retrospective Case-control study	N=108 (24 RIF/31 Control) Note: Study also included 53 RPL patients.	RIF: ≥4 good-quality embryos in ≥3 cycles; age <40. Control: Proven fertility (≥2 children); no miscarriage history.	Not assessed; morphology only.	Blood; timing not reported.	Monocyte subsets and surface chemokine receptors (CCR2, CCR5, CX3CR1); Plasma cytokines (including TGF-B1, TGF-B3, IL-8, IL-18, etc.) Assay: Multiparametric flow cytometry (for monocytes) and Luminex multiplex assay (for cytokines).	Finding: The percentage of intermediate monocytes expressing CCR5+ and the plasma levels of TGF-β3 were significantly higher in the RIF group compared to healthy controls. Diagnostic Utility: A combined multivariate model including CCR5+ intermediate monocytes and TGF-β3 yielded an AUC of 0.833 (95% CI: 0.667-0.999); Sens: 80.0%; Spec: 85.7%.
Guo et al., 2022; China (30)	Prospective cohort study	N=70 (41 RIF/29 Control)	RIF: ≥4 high-quality embryos in ≥3 cycles; age <40. Control: No adverse obstetric history; clinical pregnancy after 1st IVF/ICSI-ET.	Not assessed; morphology only (Gardner grade >4BC).	Blood (serum); mid-luteal phase prior to FET.	Cytokines including IL-2, IL-4, IL-6, IL-10, IL-17A, IFN-γ, TNF-α, TNF-β, G-CSF, and GM-CSF Assay: Multiplex immunoassay (AimPlex).	Finding: Serum IL-6 levels were significantly higher, while IL-10 and G-CSF levels were significantly lower, in the RIF group compared to controls. Diagnostic Utility: A combined predictive model (including age, endometrial thickness, IL-6, IL-10, G-CSF, and Th1/Th2 ratios) yielded an AUC of 0.94 (95% CI: 0.87-1.00), with a sensitivity of 96.55% and specificity of 87.50%.

*95% Confidence Intervals (CIs) were not reported in the primary study and were derived by the authors using the Hanley & McNeil method or Wilson score interval based on reported sample sizes.

**Table 2:** Summary of studies evaluating metabolic, protein-based, epigenetic, and genetic biomarkers for the diagnosis of RIF.

Study	Study design	Population	RIF definition & control group	Embryo ploidy status & selection	Fluid & sample timing	Biomarker(s) & assay method	Key findings & clinical utility statistics
RoyChoudhury et al., 2016; India (31)	Retrospective Case-control study	N=52 (28 RIF/24 Recurrent Implantation Success (RIS))	RIF: ≥3 failed IVF cycles; 3 high-grade embryos/cycle. Control: Recurrent Implantation Success (RIS); successful implantation every IVF-ET.	Not assessed; morphology only.	Blood (serum); implantation window.	Metabolites (Valine, Adipic acid, L-lysine, Creatine, Ornithine, Glycerol, D-glucose, Urea) and endothelial nitric oxide synthase (eNOS); Assay: Nuclear Magnetic Resonance (NMR) spectroscopy for metabolites; and enzyme immunoassay for eNOS.	Finding: Eight metabolites (Valine, Adipic acid, L-lysine, Creatine, Ornithine, Glycerol, D-glucose, and Urea) were significantly up-regulated in the serum of women with RIF compared to those with RIS. Serum eNOS levels were significantly lower in the RIF group. Diagnostic Utility: AUC values for discriminating RIF from RIS were reported for individual metabolites: Adipic acid (AUC: 0.75 [0.62–0.88]*), Urea (AUC: 0.75 [0.62–0.88]*), D-glucose (AUC: 0.74 [0.61–0.87]*), Valine (AUC: 0.74 [0.61–0.87]*), L-lysine (AUC: 0.73 [0.59–0.87]*), Creatine (AUC: 0.70 [0.56–0.84]*), Glycerol (AUC: 0.67 [0.52–0.82]*), Ornithine (AUC: 0.64 [0.49–0.79]*)
Zheng et al., 2023; China (32)	Prospective cohort study with a nested case-control analysis.	N=38 (19 RIF/19 Control)	RIF: ≥4 good-quality cleavage embryos or ≥2 blastocysts in ≥3 cycles. Control: Live birth from same cohort; matched for age, BMI, AFC.	Not assessed; morphology only.	Blood (serum); day of progesterone administration (dPA) and day 3 post-administration (d3PA) in HRT cycle.	Metabolites (Indole-3-propionic acid, beta-alanine, myristoleic acid, malic acid, indole, DL-isocitric acid, proline, and itaconic acid), analyzed as a ratio between two time points; Assay: Gas chromatography-mass spectrometry (GC-MS).	Finding: A composite model incorporating eight metabolite ratios achieved an overall accuracy of 91% for identifying women with RIF. Diagnostic Utility: Indol-3-propionic acid: AUC: 0.828 [0.69–0.96]*. Malic acid: AUC: 0.77 [0.60–0.94]*. Combined model (8 metabolites): Accuracy: 91% (AUC not numerically reported; Model Accuracy improved from 59% to 91% using dynamic ratios).
Bastu et al., 2015; Turkey (33)	Retrospective Case-control study	N=49 (26 RIF/23 Control)	RIF: ≥4 high-grade embryos in ≥3 cycles; age <40. Control: Proven fertility (≥1 birth); no infertility/abortion history; no hormonal treatment >3 months.	Not assessed; morphology only (all grade 1).	Blood (serum); days 7–9 post-LH surge (implantation window).	Mucin 1 (MUC-1), Glycodelin A (GdA); Assay: ELISA	Finding: Blood and endometrial tissue levels of MUC-1 and GdA were significantly lower in the RIF group compared to fertile controls. Diagnostic Utility: Blood MUC-1: AUC: Not Reported. Sens: 88.5% (at cutoff 0.89 ng/ml). Spec: 82.6% (at cutoff 0.89 ng/ml) Blood GdA: AUC: Not Reported. Sens: 88.5% (at cutoff 1.73 ng/ml). Spec: 87% (at cutoff 1.73 ng/ml)
Ozgu-Erdinc et al., 2020; Turkey (34)	Retrospective Case-control study	N=78 (42 RIF/36 Control)	RIF: ≥2 failed IVF cycles; age <40; no live birth history. Control: Age-matched; regular cycles; history of spontaneous live birth.	Not Reported	Blood (serum); days 1–3 of menstruation, fasting.	Fetuin-A Assay: ELISA	Finding: Serum fetuin-A levels were significantly higher in the RIF group compared to the control group. Diagnostic Utility: AUC: 0.763 (95% CI: 0.646-0.879); Sens: 85.71% (95% CI: 70.77-94.06); Spec: 66.67% (95% CI: 48.94-80.9) at a cut-off of 230.75 µg/mL.
Chen et al., 2021; China (35)	Retrospective Case-Control Study (based on bioinformatics analysis of publicly available data)	N=12 (7 RIF/5 Control) (Note: This population from dataset GSE71331/GSE71332 was used for differential expression and network analysis. The final model validation figure reports n=56 without textual explanation)	RIF: ≥4 high-quality embryos in ≥3 cycles, or ≥10 embryos total; age <40. Control: 0 failed cycles (GSE71331 dataset)	Not assessed; morphology only.	Blood (serum); implantation window.	Circulating microRNAs (hsa-miR-96-5p, hsa-miR-378e); Assay: Bioinformatics analysis of public microarray (data from GEO database).	Finding: A predictive nomogram model for RIF risk was constructed using patient age and the expression of circulating hsa-miR-96-5p and hsa-miR-378e. Diagnostic Utility: AUC: 0.865 [0.77–0.96]*.
Nardi et al., 2016; Brazil (36)	Retrospective Case-control study	N=58 (35 Implantation Failure/23 Control) for biomarker analysis N=83 (49 Implantation Failure/34 Control) for genetic analysis	RIF: ≥2 failed ART transfers. Control: Proven fertility (≥2 pregnancies); no gestational complications.	Not Reported	Blood (serum); Not Reported. (Note: germline DNA analysis; sample timing not critical.)	Total soluble HLA-G (sHLA−G tot) and Extracellular Vesicle-derived soluble HLA-G (sHLA−G EV); Assay: ELISA	Finding: Women with implantation failure had significantly higher levels of total soluble HLA-G (sHLA−G tot) and a higher proportion had elevated vesicular HLA-G (sHLA−G EV) compared to fertile controls. Diagnostic Utility: For total sHLA-G (sHLA−G tot) at a cutoff of 6.125 ng/mL. For total sHLA-G (sHLA−G tot): AUC: 0.69 [0.58–0.80]*; Sens: 85.4%; Spec: 53.1%. For vesicular sHLA-G (sHLA−GEV) at a cutoff of 2.47 ng/mL: Sens: 94.3%; Spec: 44.0%.

*95% Confidence Intervals (CIs) were not reported in the primary study and were derived by the authors using the Hanley & McNeil method or Wilson score interval based on reported sample sizes.

**Table 3:** Summary of studies evaluating prognostic biomarkers in blood and diagnostic biomarkers in uterine fluid.

Study	Study design	Population	RIF definition & control group	Embryo ploidy status & selection	Fluid & sample timing	Biomarker(s) & assay method	Key findings & clinical utility statistics
Piekarska et al., 2023; Poland (37)	Retrospective Case-Control Study	N=254 (187 RIF/67 Control)	RIF: Average 3 failed transfers; 3 good-quality embryos/transfer. Control: ≥1 healthy child after natural conception; no miscarriage or immunological/endocrinological disease.	Not assessed; morphology only.	Blood (plasma); before ET and 11–15 days post-ET.	IFN-γ, IL-10, BDNF, LIF, soluble TNFR1 (sTNFR1), VEGF-A; Assay Method: Luminex multiplex immunoassay (ProcartaPlex assay)	Finding: Plasma levels of sTNFR1 were significantly lower in patients who experienced a lack of pregnancy compared to those who achieved pregnancy. Prognostic Utility: For predicting pregnancy vs. no pregnancy/miscarriage in IVF patients using sTNFR1: AUC: 0.66 [0.57–0.75]*; Sens: 62.7%; Spec: 62.2%. VEGF-A: AUC: 0.69; Sens: 65.5%; Spec: 65.8%. Also, plasma VEGF-A level above 43.28 pg/mL was associated with a greater risk of miscarriage or failed transfer.
Piekarska et al., 2021; Poland (38)	Retrospective Case-Control Study	N=881 (496 total IVF patients, of which 283 had RIF/385 Fertile Control)	RIF: ≥4 good-quality embryos in ≥3 cycles; age <40. Control: ≥1 healthy child after natural conception.	Not assessed; morphology only.	Blood (plasma); before ET and at hCG testing.	Endoplasmic Reticulum Aminopeptidase 1 and 2 (ERAP1) and (ERAP2). Assay: Sandwich ELISA kit.	Finding: Plasma ERAP2 levels were significantly higher in patients who miscarried after IVF-ET compared to those who had a successful pregnancy. Elevated ERAP2 levels above a threshold of approximately 2.9 ng/ml were identified as a risk factor for miscarriage. Prognostic Utility: For predicting miscarriage (vs. successful pregnancy) in IVF patients using plasma ERAP2: AUC: 0.64 [0.55–0.73]*; Sens: 65.38%; Spec: 64.39%; Sens: 65.38%; Spec: 64.39% (Threshold: 2.92 ng/ml).
Huang et al., 2021a; China (39)	Prospective cohort study	N=38 (38 uRIF/0 Control)	RIF: ≥3 IVF failures; ≥1 high-quality embryo/cycle; unexplained RIF (uRIF). Control: Not applicable (no control group).	Not assessed; morphology only.	Blood; mid-luteal phase prior to ET.	Percentage of NKG2D-positive (NKG2D+) γδ-T cells in total lymphocytes. Assay: Flow Cytometry	Finding: The percentage of peripheral blood NKG2D+ γδ-T cells in lymphocytes was significantly higher in uRIF patients who subsequently failed to achieve a clinical pregnancy compared to those who were successful. Prognostic Utility: For predicting clinical pregnancy failure: AUC: 0.774 (95% CI: 0.597-0.951); Sens: 92.3%; Spec: 66.7% (at a cut-off value of 3.24%).
Huang et al., 2021b; China (40)	Prospective cohort study	N=75 (54 uRIF/21 Control)	RIF: ≥3 failed IVF cycles; high-quality embryos; uRIF. Control: No infertility history; regular cycles; ≥1 successful pregnancy.	Not assessed; morphology only.	Whole blood; mid-luteal phase prior to FET.	Cytotoxic granules (Perforin, Granzyme B, Granulysin) in γδ-T cells and NK cells. Assay: Flow cytometry.	Findings: The percentage of GrB+ γδ-T cells in lymphocytes was significantly higher in uRIF patients with clinical pregnancy failure than those with a successful pregnancy. The combined GrB+ γδ-T and GrB+ NK cell indicator synergistically improved predictive accuracy. Prognostic Utility: For predicting clinical pregnancy failure using GrB+ γδ-T cells alone: AUC: 0.661 (95% CI: 0.516-0.807); Sens: 64.3%; Spec: 69.2% (at >3.03% cut-off). For combined GrB+ γδ-T and GrB+ NK cells: AUC: 0.717.
Li et al., 2023; China (41)	Retrospective Cohort followed by a Prospective Cohort Study	N=546 (433 in retrospective part; 113 in prospective part) (546 RIF/0 Control)	RIF: ≥2 failed cycles (≥4 cleavage embryos or ≥2 blastocysts); uRIF. Control: Not applicable (no control group).	Not assessed; morphology only.	Blood (plasma); day of embryo transfer	D-dimer Assay: ACL-Advance automatic coagulation analyzer (ACL-TOP-700)	Finding: Plasma D-dimer levels were significantly lower in uRIF patients who achieved a live birth compared to those who did not. In the prospective cohort, the live birth rate was significantly higher in patients with D-dimer levels ≤0.22 mg/L compared to those with levels >0.22 mg/L. Prognostic Utility: For predicting live birth within the RIF cohort: AUC: 0.806 (95% CI: 0.763-0.848); Sens: 71.3%; Spec: 81.2% (at a cutoff value of 0.22 mg/L)
Kasvandik et al., 2020; Estonia & Sweden (42)	Retrospective Case-control study	N=47 (29 RIF/18 Control) Discovery cohort: 6 fertile controls Validation cohort: 12 controls with male/tubal factor infertility and 29 RIF patients	RIF: ≥3 failed IVF cycles with good-quality embryos, or ≥10 embryos total. Control: Composite; proven fertile women + first IVF cycle for male/tubal-factor infertility.	Not assessed; morphology only.	Uterine fluid; early secretory phase (ESE: LH + 1 to +3) and/or mid-secretory phase (MSE: LH + 7 to +9).	A 4-protein panel consisting of Progesterone Receptor (PGR), Nicotinamide N-methyltransferase (NNMT), Solute Carrier Family 26 Member 2 (SLC26A2), and Lipocalin-2 (LCN2). Discovery: Liquid chromatography tandem-mass spectrometry (LC-MS/MS). Validation: Targeted mass spectrometry (parallel reaction monitoring).	Finding: The uterine fluid protein profile of women with RIF in the mid-secretory phase (MSE) was similar to that of fertile controls in the early secretory phase (ESE), suggesting a displaced window of implantation. Diagnostic Utility: For distinguishing RIF MSE from fertile control MSE using a 4-protein panel: PGR (Upregulated); NNMT, SLC26A2, LCN2 (Downregulated). AUC: Not Reported; Sens: 96.6%; Spec: 91.7%

*95% Confidence Intervals (CIs) were not reported in the primary study and were derived by the authors using the Hanley & McNeil method or Wilson score interval based on reported sample sizes.

### 3.3 Quality assessment of included studies

**Figure 3:**
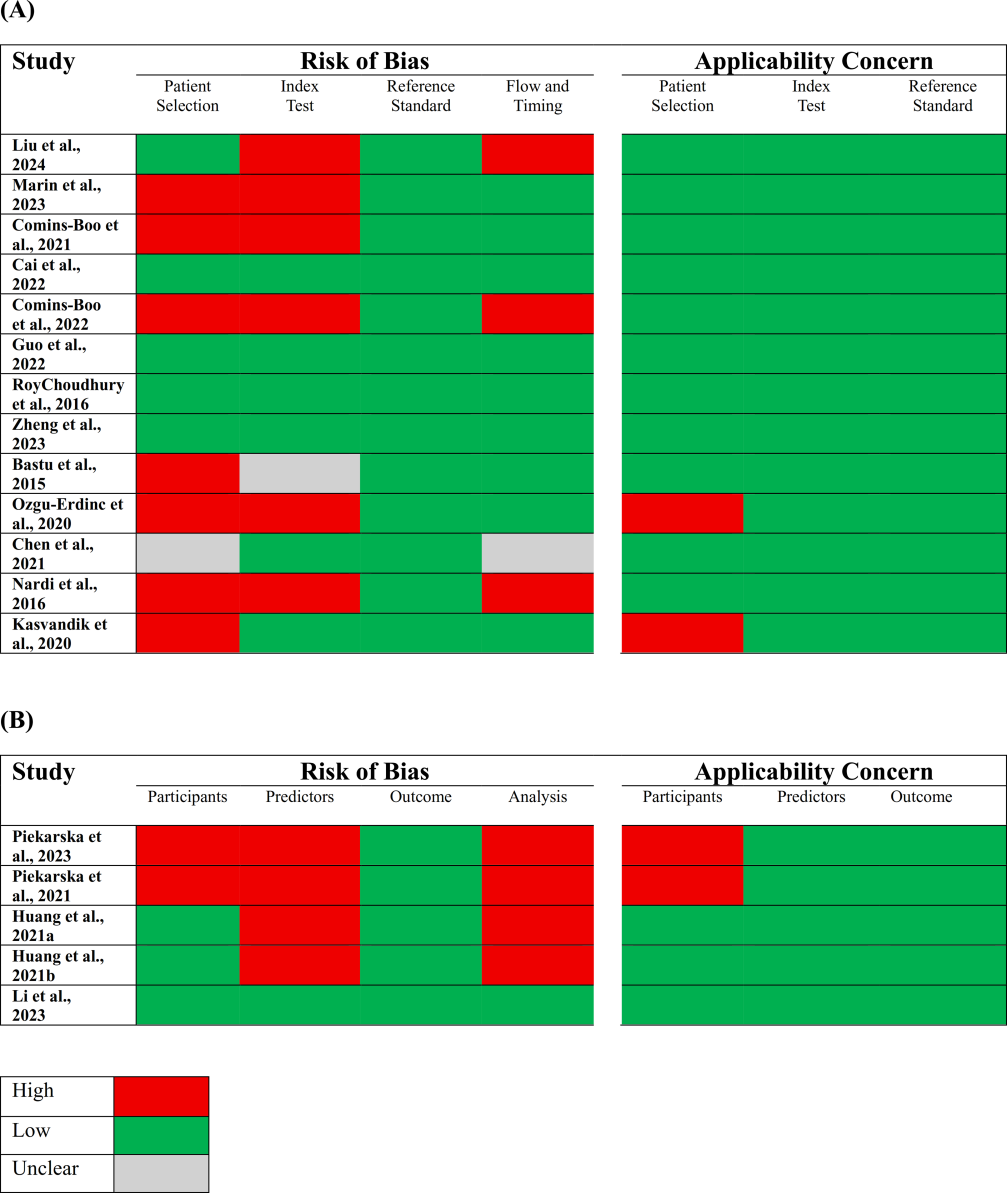
**(A)** Risk of bias and applicability concerns summary for diagnostic studies, using QUADAS-2 tool. **(B)** Risk of bias and applicability concerns summary for prognostic studies, using the PROBAST tool.

The methodological quality of the 18 included studies was evaluated using the QUADAS-2 and PROBAST frameworks (**Figure 3**). Overall, the methodological quality assessment of available evidence was limited. Only 5 studies were judged to have a low risk of bias across all domains, while 10 exhibited a high risk in at least two domains, and 3 raised moderate concerns.

Diagnostic evidence (n=13) was undermined by significant flaws in patient selection. While seven studies (54%) employed clinically relevant controls (infertile women with IVF success), nearly half (6/13, 46%) relied on inappropriate “two-gate” designs (a study design that compares patients with a condition against a healthy population rather than a clinically relevant comparator group) comparing RIF patients against healthy fertile women, a design known to overestimate diagnostic performance. Furthermore, significant intra-group heterogeneity within RIF cohorts was a major concern. Many studies amalgamated patients with disparate clinical histories, varying numbers of failed cycles, embryos transferred, and etiologies, into a single “RIF” group. This grouping introduces confounding and limits generalizability (5). In the Index Test domain, issues included variable sample timing and a lack of predefined biomarker thresholds, introducing a high risk of data-driven threshold selection. The Reference Standard domain was compromised by the use of vague and inconsistent RIF definitions across studies. Critically, none of the studies restricted inclusion to euploid embryo transfers, introducing embryonic aneuploidy as a significant confounding variable.

Prognostic evidence (n=5) was similarly limited, with only one study demonstrating a low risk of bias. The most pervasive issue was the *post-hoc* selection of biomarker thresholds combined with a lack of independent model validation, severely limiting the generalizability of findings. Further concerns included underpowered analyses and failure to account for clinical confounders.

### 3.4 Diagnostic utility of blood biomarkers

A summary of the diagnostic performance of all identified biomarkers is presented in **Figure 1**. The forest plot illustrates a distinct performance hierarchy, where multi-marker panels consistently outperform single analytes.

#### 3.4.1 Immunological markers

Evaluations of routine hematological parameters showed limited diagnostic utility. A large retrospective case–control study reported significantly reduced levels of platelets, plateletcrit, and lymphocytes in women with RIF compared to fertile controls. However, these parameters failed to accurately distinguish RIF patients from fertile controls, with lymphocyte count yielding an AUC of 0.577 (24). Similarly, elevated levels of monocytic myeloid-derived suppressor cells (M-MDSCs; innate immune cells with potent immunosuppressive activity) in RIF were observed, with an AUC of 0.690 (25). One study indicated that increased expression of surface markers NKp30 and TIGIT on cytotoxic NK cells in RIF patients was identified, with NKp30 achieved AUC of 0.80 (**Table 1**) (26). However, a key methodological limitation across these three studies was the absence of cycle-phase stratification at the time of blood sampling, an omission that potentially introduces confounding due to hormonal variation in circulating lymphocyte numbers (27).

Recent studies have shifted toward multiplexed immune profiling. A diagnostic model was constructed based on three blood biomarkers (Treg, Tfh17, and early inhibitory NK cells), which demonstrated an AUC of 0.90 for distinguishing RIF patients from controls (28). Similarly, a combination of a cellular marker (CCR5 expression on intermediate monocytes) with a soluble cytokine (TGF-β3) achieved an AUC of 0.83 (29). Another study extended this approach by integrating multiple serum cytokines (including IL-6, IL-10, G-CSF, and Th1/Th2 ratios) with clinical parameters (age and endometrial thickness). Their multivariable model yielded an AUC of 0.94 (sensitivity 96.55%; specificity 87.5%) (**Table 1**) (30). While Cai and Guo presented models with low risk of bias, the findings by Comins-Boo et al. (2022) should be interpreted with caution due to high risk of bias.

#### 3.4.2 Metabolic markers

Metabolomic profiling revealed systemic disturbances in women with RIF. In a case-control study, serum metabolic profiles of RIF patients were compared against a control group of women with recurrent implantation success. Eight metabolites were significantly elevated in RIF, with adipic acid and urea demonstrating the highest individual diagnostic accuracy each showing an AUC of 0.75 (31). A recent prospective analysis assessed temporal changes in serum metabolite concentrations between the day of progesterone administration and the third day after administration. The study demonstrated that using dynamic metabolite ratios, rather than static levels, significantly enhanced diagnostic performance, improving overall model accuracy from 59% to 91%. Within this eight-marker composite model, Indole-3-propionic acid and malic acid were identified as key predictors, achieving individual AUCs of 0.828 and 0.770, respectively (**Table 2**) (32). Both studies had a low risk of bias (**Figure 3A**) and controlled for confounders, including body mass index (BMI) and pre-existing metabolic conditions.

#### 3.4.3 Implantation-associated proteins

Several studies have evaluated circulating proteins linked to endometrial receptivity as RIF biomarkers. Significantly reduced serum concentrations of Mucin-1 (MUC-1) and Glycodelin A (GdA) in RIF patients were reported. These markers showed sensitivity (88.5%) and specificity (MUC-1: 82.6%, GdA: 87.0%) and correlated well with endometrial tissue levels in relatively small cohorts without external validation (33). Additionally, fetuin-A was found elevated in RIF patients, with an AUC of 0.763 (34).

#### 3.4.4 Epigenetic markers

Circulating microRNAs have emerged as a novel class of minimally invasive biomarkers. A bioinformatics-driven diagnostic model incorporating two serum miRNAs (miR-378e and miR-96-5p) alongside patient age yielded an AUC of 0.865 (**Table 2**) (35). However, the promising findings of this study should be interpreted with caution, as the study was noted to have some concerns regarding potential bias (**Figure 3A**).

#### 3.4.5 Genetic markers

The contribution of inherited genetic variation to RIF susceptibility has been explored through the analysis of specific polymorphisms. One study investigated the HLA-G 14-bp insertion/deletion polymorphism, finding the deletion allele to be more prevalent among RIF patients and associated with elevated serum soluble HLA-G levels. Despite a high sensitivity (85.4%), specificity was low (53.1%) (**Table 2**) (36). This study had a high risk of bias (**Figure 3A**).

### 3.5 Prognostic utility of blood biomarkers

Five studies investigated the prognostic value of blood biomarkers in predicting RIF clinical outcomes. Two studies investigated soluble immune-related proteins as potential biomarkers for predicting pregnancy outcomes. In one study, soluble tumor necrosis factor receptor 1 (sTNFR1; AUC = 0.66) and vascular endothelial growth factor A (VEGF-A; AUC = 0.69) were identified as favorable predictors of pregnancy in women with RIF (37). Another study conducted in a mixed IVF cohort reported that endoplasmic reticulum aminopeptidase 2 (ERAP2) was negatively associated with clinical pregnancy (ERAP2: AUC = 0.64) (38). Both studies had a high risk of bias (**Figure 3B**), primarily due to study design and patient selection. Another study reported that an increased proportion of circulating NKG2D^+^ γδ T cells predicted pregnancy failure (AUC = 0.774) (39). Another investigation found a combined prognostic model using both Granzyme B-expressing (GrB+) γδ-T cells (a subset of T lymphocytes expressing the gamma-delta T cell receptor, involved in innate immune surveillance) and GrB+ NK cells achieved an AUC of 0.717 to predict clinical pregnancy (40). Beyond immunology, an investigation with low risk of bias found that plasma D-dimer levels >0.22 mg/L on the day of embryo transfer were associated with adverse outcomes (AUC = 0.806) (41).

### 3.6 Biomarkers in uterine fluid

This review identified a single study evaluating biomarkers in uterine fluid among women with RIF. In this proteomics-driven discovery, the mid-secretory phase endometrial secretome (the ensemble of proteins actively secreted by a tissue or cell type into the surrounding fluid environment) was assessed, and a four-protein biomarker panel [progesterone receptor (PGR), nicotinamide N-methyltransferase (NNMT), solute carrier family 26 member 2 (SLC26A2), and lipocalin-2 (LCN2)] was identified that distinguished RIF patients from fertile controls, with 96.6% sensitivity and 91.7% specificity. Specifically, the study found that PGR was upregulated, while NNMT, SLC26A2, and LCN2 were downregulated in the uterine fluid of women with RIF compared to fertile controls (**Table 3**) (42). Notably, this study utilized external validation cohorts, albeit with a relatively small sample size. However, risk of bias was rated “some concerns,” primarily because the study included only patients who were diagnosed with either male-factor or tubal infertility causes of RIF, which limits the generalizability of the findings to a broader RIF population (**Figure 3A**).

### 3.7 Biomarkers in menstrual fluid

No eligible studies evaluating biomarkers in menstrual fluid for the diagnosis or prognosis of RIF were identified.

## 4 Discussion

This systematic review identified a clear performance hierarchy among fluid-based biomarkers for RIF. Multi-marker models consistently demonstrated higher diagnostic performance than single analytes, with several integrated blood-based panels reporting AUCs above 0.90 in discovery cohorts. In addition, a four-protein signature identified in uterine fluid showed promising diagnostic performance. Nonetheless, prognostic evidence remains limited. Only five studies evaluated clinical outcomes, and most biomarkers showed modest predictive performance. Further, the current literature is dominated by blood-based studies, whereas uterine fluid biomarkers remain underexplored and no eligible studies were identified for menstrual fluid biomarkers.

Within the scope of this review on fluid-based biomarkers, immune dysregulation emerged as the most-studied pathogenic mechanism in RIF. Multiplex immunologic models demonstrate promising diagnostic performance (AUC = 0.833–0.94), suggesting the possibility of systemic immune imbalance rather than single-cytokine aberrations. These findings can support the concept of RIF as a heterogeneous, immune-defined syndrome characterized by complex immune dysregulation. However, a recent study reported a Th2-biased serum profile for RIF alongside a pro-inflammatory Th1-dominant follicular fluid signature (43), suggesting systemic immune profiles may not fully capture local uterine immune profiles. Consistently, major clinical practice guidelines discourage the clinical use of blood immune markers due to limited concordance with uterine immune profiles and limited assay validation (44). This discrepancy highlights the challenges in translating systemic immune signatures into clinically actionable insights, emphasizing the need for further validation and integration with local endometrial data. Interpretation of immune biomarker performance is further limited by the control groups used in several studies, which compared RIF patients with naturally fertile women rather than ART patients with successful implantation. This two-gate design likely inflated diagnostic performance, as naturally fertile women represent a biologically distinct population and may not reflect the true discriminatory value of these biomarkers in clinically relevant ART settings.

Our findings align with previous reports describing altered circulating metabolomic profiles in RIF, particularly involving amino acid metabolism. A prior systematic review (45) based on a limited number of studies, reported similar trends but found the evidence to be inconclusive. More recent studies have expanded this field by incorporating temporally resolved and dynamic metabolomic assessments, with multivariate models demonstrating high diagnostic performance (up to 91%) compared with single-analyte approaches. These findings suggest that dynamic metabolic responsiveness to hormonal cues may better reflect underlying endometrial dysfunction than static metabolite measurements (32). Beyond metabolic profiling, the observed systemic deficiency in the pro-implantation protein MUC-1 aligns with the broader hypothesis that impaired endometrial signaling may be reflected in circulating biomarkers in RIF. Consistently, reduced serum kisspeptin levels have been associated with implantation failure, particularly in women with unexplained infertility (46, 47). Yet, circulating protein biomarkers remain underrepresented in prior reviews (48), highlighting an area that warrants further investigation.

Emerging evidence suggests that circulating microRNAs may serve as minimally invasive biomarkers in RIF, given their roles in regulating gene expression and their stability in peripheral blood (49). However, current evidence for their application in RIF remains limited and largely derived from exploratory modeling and bioinformatic analyses. Multi-miRNA classifiers have demonstrated encouraging discriminatory performance in preliminary studies, and some diagnostic platforms based on circulating microRNA signatures have been proposed in the broader reproductive medicine literature (e.g., the MIRA Test) (50, 51). Nevertheless, these approaches require rigorous independent validation in well-characterized prospective cohorts before their clinical utility in RIF can be established.

Beyond diagnosis, the prognostic utility of blood-based biomarkers in predicting implantation outcomes within RIF cohorts remains considerably more limited. While D-dimer showed good performance, most individual markers had modest accuracy, consistent with findings for pre-implantation CRP in forecasting ART failure (52). However, integrating multiple cellular markers appears to enhance prognostic power. For example, combining Granzyme B–expressing γδ-T and NK cells provides stronger prediction of clinical pregnancy failure than assessing either population alone (40). Furthermore, recent prospective research has evaluated other promising prognostic markers from the broader ART literature and within RIF cohorts. For instance, while meta-analyses in general ART populations link low luteal progesterone to poorer outcomes (53), a prospective controlled study showed this trend specifically in RIF patients, finding lower mid-luteal levels compared to controls even under standardized support (54). The role of vitamin D remains debated in broader ART literature. While one meta-analysis (55), found a positive association with live birth outcomes, a subsequent analysis using rigorous sensitivity analyses (56) found no such link. Nevertheless, the same prospective controlled study provided crucial evidence by demonstrating that women with RIF have significantly lower mean serum vitamin D levels than controls (54), suggesting systemic hormonal and metabolic deficiencies may present in the RIF population. In a large 2025 cohort, Wang et al. identified serum anti-Müllerian hormone (AMH) as the predictor of RIF, with low AMH levels conferring the greatest risk. By integrating blood-based biomarkers (AMH, FSH, and testosterone) with clinical factors (chronic endometritis and BMI), this model achieved an AUC of 0.78 and supports the multifactorial nature of RIF (57).

Evidence on uterine fluid-based biomarkers in RIF remains scarce (48). A single eligible study evaluated a multi-marker protein panel, which suggested that the endometrium in RIF patients may persist in a pre-receptive, early secretory–like state, consistent with impaired endometrial maturation (42). However, uterine fluid represents a biologically relevant compartment because it directly reflects the endometrial secretory environment at the maternal–embryo interface (58). A seminal prospective cohort study by Boomsma et al. (2009) first demonstrated that a specific cytokine profile in endometrial secretions, higher TNF-α and lower IL-1β, predicted clinical pregnancy. Also, this profile added predictive value beyond embryo quality, providing early evidence that the endometrium independently influences implantation outcomes (59). Recent reviews also emphasize uterine fluid’s potential as a biologically rich, less-invasive source for diagnostic innovation (60). Recent advances suggest potential for multi-omic and microbiomic uterine fluid profiling, and individual studies point to the diagnostic value of proteins, cytokines, extracellular vesicle content, and fluid microbiome (17, 58, 61). However, the field is hindered by small, heterogeneous studies often lacking clinical validation.

A key finding of this review is the absence of studies investigating menstrual fluid in RIF biomarker research, even though recent studies highlight its theoretical potential (49). Uniquely, menstrual fluid represents a non-invasive ‘liquid biopsy’ of the shedding functionalis layer, theoretically offering a direct window into the endometrial immunome that systemic blood may not provide (62, 63). Preliminary data from broader infertility cohorts suggest that multi-analyte protein and immune cell signatures in menstrual fluid may carry some diagnostic signal (18, 20, 64–68). In this context, a recent preliminary study used EV proteomics to classify unexplained infertility subtypes with reporting AUCs of ~1.0 (20). Moreover, cytokine assays of menstrual effluent have demonstrated promising accuracy in diagnosing chronic endometritis (AUC = 0.989) (18). However, RIF-specific evidence remains extremely limited, highlighting a substantial gap in the literature. This likely reflects methodological challenges, including variability in sampling timing and heterogeneity in cellular and molecular composition (69). Taken together, the clinical utility of menstrual fluid biomarkers in RIF remains unestablished. Nevertheless, its non-invasive nature and capacity to capture endometrial and immune dynamics can support prioritization in future research.

### 4.1 Strengths and limitations

This systematic review provides a comprehensive evaluation of fluid-based biomarkers for RIF across systemic and local biological compartments. To our knowledge, no previous systematic review has comparatively evaluated biomarkers across blood, uterine fluid, and menstrual fluid within a single analysis, highlighting the disparity in the current research landscape. Methodological rigor was ensured through adherence to PRISMA guidelines, protocol pre-registration, and the application of an additional TRACT-based screening step to identify studies with potential data integrity concerns. Diagnostic performance across biomarkers was summarized using forest plots, and risk of bias was assessed using design-specific tools (QUADAS-2 for diagnostic studies and PROBAST for prognostic studies).

Despite these strengths, some limitations arise from the underlying primary literature. A major limitation across all included studies was the absence of ploidy control. Since embryonic aneuploidy is an important cause of implantation failure, including aneuploid transfers likely misclassified embryo-driven failures as endometrial dysfunction, thereby limiting the ability of maternal biomarkers to accurately identify true endometrial pathology and potentially inflating their apparent performance. Additionally, there was a marked variability in RIF definitions across included studies, which likely contributes to the differences in biomarker performance observed throughout this review. This inconsistency contrasts with recent ESHRE guidelines on RIF (44), which have specifically emphasized the need for standardized diagnostic criteria. Variability in biomarker assays and sampling protocols further limited comparability and precluded quantitative meta-analysis. In addition, most biomarker models were derived from single-center discovery cohorts without internal or independent external validation, increasing the risk of optimistic performance estimates.

### 4.2 Clinical implications and perspectives

The findings of this review highlight increasing interest in minimally invasive biomarkers for assessing endometrial function in RIF, particularly given the limitations of invasive tissue-based diagnostic approaches (13, 70). Multi-marker biomarker models demonstrated higher diagnostic performance than single analytes; however, their clinical implementation requires robust external validation. Blood-based multi-marker panels, if further studied and validated, may have the potential to represent practical and scalable diagnostic tools, enabling minimally invasive assessment within routine ART cycles.

A major research priority is methodological standardization. Consensus on RIF definitions, biomarker panels, sampling timing, and laboratory protocols is essential to enable meaningful comparison across studies and facilitate clinical translation. As highlighted in recent analyses (5), the absence of standardized RIF diagnostic criteria has contributed to a heterogeneous evidence base that remains difficult to synthesize and interpret.

Future studies should therefore be prospective, multicenter, and adequately powered, with predefined analytical thresholds and adherence to TRIPOD (Transparent Reporting of a multivariable prediction model for Individual Prognosis Or Diagnosis) guidelines for biomarker model validation. Prioritizing cohorts undergoing euploid embryo transfer would further reduce embryonic aneuploidy as a confounding factor and allow clearer evaluation of maternal contributions to implantation failure. Beyond diagnostic accuracy, future research should also aim to identify biologically distinct endotypes (biologically distinct disease subtypes defined by underlying molecular mechanisms) that could support targeted therapeutic strategies and randomized trials in stratified patient populations.

In addition to blood-based biomarkers, uterine and menstrual fluids represent promising but underexplored sources of diagnostic information. Blood-based biomarkers offer practical advantages in clinical settings, including minimal invasiveness, standardized collection protocols, and ready integration into routine ART care. Emerging evidence suggests that the circulating proteome may partially reflect endometrial tissue function. A recent large-scale plasma proteomic study identified 198 proteins that vary systematically across menstrual cycle phases, many of which are enriched in endometrial epithelial and stromal cell types, suggesting that cycle-dependent blood proteins may carry biologically relevant endometrial signals (71). Nevertheless, blood-based markers may still lack the specificity needed to accurately capture local endometrial dysfunction at the maternal-embryo interface. In contrast, uterine fluid directly reflects the endometrial secretory environment and may therefore offer greater biological proximity to the implantation niche. However, its collection requires intrauterine sampling, which is more invasive and less practical for routine clinical use. Taken together, these complementary strengths and limitations suggest that future diagnostic approaches may benefit from integrating systemic and local biomarker profiles, potentially enabling more refined assessment of implantation biology and supporting personalized management strategies for RIF.

## 5 Conclusion

Fluid-based biomarkers, particularly multi-marker models, may have potential as diagnostic tools in RIF. However, current evidence is largely derived from exploratory and heterogeneous studies, limiting immediate clinical applicability. Future progress will require prospective validation of multi-marker models within standardized RIF cohorts that adequately control for embryonic euploidy. Expanding research into biologically proximal uterine and menstrual biofluids may provide more direct insights into endometrial function and support the development of more individualized approaches to clinical care in RIF.

## Data Availability

The original contributions presented in the study are included in the article/**Supplementary Material**. Further inquiries can be directed to the corresponding author.
