# Supplementary material for: Clinical potential of fluid-based biomarkers for recurrent implantation failure: a systematic review
Source: Front Endocrinol (Lausanne). 2026 Jun 12;17:1854111. doi: 10.3389/fendo.2026.1854111 (PMC13303133; doi:10.3389/fendo.2026.1854111)
Supplement: Supplementary file 1 [file DataSheet1.docx]

Supplementary Material

# Full search strategy:

# The search strategy was built around three core concepts, combining both keywords and MeSH terms using the AND operator:

# 1. Condition: Terms related to recurrent implantation failure and endometrial receptivity (e.g., "implantation failure", "recurrent implantation failure", "repeated implantation failure", "unexplained infertility", "endometrial receptivity", etc.) using the OR operator.

# 2. Fluid Type: Terms describing relevant biological samples (e.g., "blood", "serum", "plasma", "uterine fluid", "uterine secret", "uterine flush", "uterine lavage", "endometrial fluid", "endometrial lavage", "endometrial secret", "menstrual fluid", "menstrual effluent", "menstrual plasma", "body fluid", etc.) using the OR operator.

# 3. Biomarkers: A wide range of terms referring to biological markers (e.g., "biomarker", "marker", "cytokine", "hormone", "extracellular vesicles", "vesicle", " exosomes " "microRNA", " transcription factors ", "metabolome", "vitamins", "growth factor", " non-coding rna", "circulating factor", etc.) using the OR operator.

# These three categories were combined using the formula:

# (1) AND (2) AND (3)
